# Calculation and Visualization of Atomistic Mechanical Stresses in Nanomaterials and Biomolecules

**DOI:** 10.1371/journal.pone.0113119

**Published:** 2014-12-11

**Authors:** Andrew T. Fenley, Hari S. Muddana, Michael K. Gilson

**Affiliations:** Skaggs School of Pharmacy and Pharmaceutical Sciences, University of California San Diego, La Jolla, California, 92093, United States of America; Politecnico di Milano, Italy

## Abstract

Many biomolecules have machine-like functions, and accordingly are discussed in terms of mechanical properties like force and motion. However, the concept of stress, a mechanical property that is of fundamental importance in the study of macroscopic mechanics, is not commonly applied in the biomolecular context. We anticipate that microscopical stress analyses of biomolecules and nanomaterials will provide useful mechanistic insights and help guide molecular design. To enable such applications, we have developed Calculator of Atomistic Mechanical Stress (CAMS), an open-source software package for computing atomic resolution stresses from molecular dynamics (MD) simulations. The software also enables decomposition of stress into contributions from bonded, nonbonded and Generalized Born potential terms. CAMS reads GROMACS topology and trajectory files, which are easily generated from AMBER files as well; and time-varying stresses may be animated and visualized in the VMD viewer. Here, we review relevant theory and present illustrative applications.

## Introduction

The rapidly expanding fields of mechanochemistry [1]–[5] and mechanobiology [6]–[13] require methods of defining and computing the mechanical properties of molecules at the atomistic level. The fundamental mechanical concept of stress is likely to be particularly useful for understanding structure-function relations in biomolecular systems like allosteric proteins, molecular motors, and mechanosensitive channels, as well as in nanoscale systems, like various graphene constructs. There is thus a need for computational tools to extract information about stress from molecular simulations.

The theory connecting macroscopic stress to microscopical forces and configurations is considered in prior works [14]–[22], and these concepts have been applied to molecular simulation data in order to analyze mechanical stress in several molecular systems. An early example is Yamato and co-workers' dynamical stress analysis of a “protein quake” in photoactive yellow protein and important follow-up work on the system [23], [24]. Other examples include applications of atomistic stress analysis to understand barriers in the dissociation pathways of high-affinity host-guest systems [25], [26], mechanical stresses in proteins in liquid and glass states [27], and stresses in lipid membranes [28] and lipid bilayers [29]. However, software to carry out similar analyses on existing simulation data is still not generally available. One post-processing tool, Force Distribution Analysis [30], [31], provides valuable information that is similar in spirit to atomistic stresses and has been applied in a variety of biophysical [32]–[35] nanomaterial [36] contexts. It is worth remarking, however, that it does not distinguish between regions of tension and compression. The widely used simulation program LAMMPS [37], [38] provides for on-the-fly calculation of atomistic stresses and is often used for simulation of materials. However, while there are some applications of LAMMPS for biomolecular simulations [39], [40], the biomolecular simulation community typically uses other software packages, such as GROMACS [41]–[43], CHARMM [44], [45], NAMD [46], GROMOS [47], and AMBER [48].

Here, we describe a new software package that computes atomistic stresses for MD simulation outputs generated by various biomolecular simulation codes. Natively, the software directly supports GROMACS file formats. However, we provide a protocol for converting simulation data from AMBER into the supported formats. The software is available in the GitHub repository (https://github.com/afenley/CAMS) and is released under the GPL version 2 open source license. As a demonstration of the software, we apply it to an equilibrium simulation of the protein BPTI and to nonequilibrium simulations of graphene nanostructures.

## Methods

### Calculation of atomic virial stresses from simulation snapshots

Mechanical stress is properly a macroscopic quantity, which can be computed in terms of microscopical (atomistic) forces and configurations, as detailed in theoretical work cited above. It is most rigorously defined for objects that are large and homogeneous enough that the local stresses can be meaningfully averaged over a characteristic volume containing many atoms. However, useful insights can be gained by considering the stress to be a quantity that varies within a heterogeneous nanoscale object, such as a protein. References provided above discuss various approaches to defining local stress; here, we use one of the simpler approaches which is to compute the virial stresses on individual atoms.

We write the stress tensor at atom *i* of a molecule in a given configuration as [17], [19]:



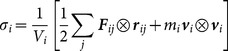
(1)


Here, 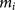, 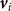, and 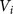 are, respectively, the mass, velocity, and characteristic volume of the atom; 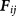 is the force acting on the *i*th atom due to the *j*th atom; and 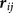 is the distance vector between atoms *i* and *j*. Here *j* ranges over atoms that lie within a cutoff distance of atom *i* and that participate with atom *i* in a non-bonded, bond-stretch, bond-angle or dihedral force term. For the analysis presented here, the cutoff distance is set to 10 Å.

The characteristic volume is normally taken to be the volume over which local stress is averaged [17], and it is required that the characteristic volumes satisfy the condition, 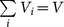 [22], where 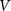 is the total simulation box volume. The characteristic volume of a single atom is not unambiguously specified by theory, so we make the somewhat arbitrary decision to set the characteristic volume to be equal per atom; i.e., the simulation box volume divided by the number of atoms, 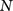: 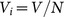. If the system has no box volume (e.g. coordinates from trajectories using implicit solvent), then each atom is assigned the volume of a carbon atom. Either way, the characteristic volumes are treated as constant over the simulation. Note that the time average of the sum of the atomic virial stress over all atoms is closely related to the pressure of the simulation.

Our chief interest is to analyze the atomistic contributions to the virial within the local coordinate system of each atom as it moves, so the stresses are computed in the local (i.e., Lagrangian) frame of reference. In this case, Equation (1) is further simplified to [49],



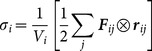
(2)


Equation (2) is directly applicable to existing simulation data where atomic velocities were not stored with the atomic coordinates. Nevertheless, the CAMS software package can, as an option, include the second term in Equation (1) if the simulation output includes velocity information.

Although Eq. 2 is straightforward to apply in the case of a purely pairwise potential, it is also applicable to more general many-body potentials [50], [51], such as bond-angles and torsions that arise in classical molecular simulations. As previously described [24], one may decompose the atomic forces (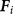) into pairwise contributions (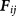) using the chain rule of differentiation:
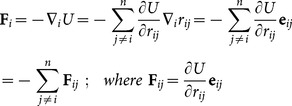



Here 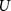 is the potential energy, 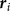 is the position vector of atom *i*, 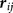 is the vector from atom *j* to *i*, and 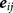 is the unit vector along 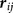. Recently, Ishikura et al. [24] have derived the equations for pairwise forces of angle and torsional potentials that are commonly used in classical force-fields. Note that, for torsional potentials whose phase angle is not 0 or π, the stress contribution contains a ratio of sine functions that is singular for certain values of the torsion angle. (For phase angles of 0 or π, the singularity can be removed by re-expressing the ratio of sine functions as a series of cosines.) However, this singularity does not pose a problem in the present study, as the force field torsion parameter values used here all have phase angle values of 0 or π. In addition, we have derived the formulae for stress contributions associated with the Onufriev-Bashford-Case (OBC) generalized Born implicit solvation model [52]. The formulae for the principal stress contributions from the various supported potential terms are summarized in Table 1.

**Table 1:** Formulae for mean principal stress (negative hydrostatic pressure) associated with potential terms in common classical force-fields.

Potential	Energy function	Principal stress
**Bond**	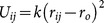	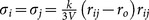
		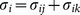
		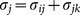
		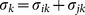
**Angle**	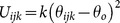	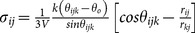
		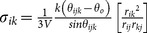
		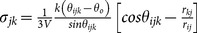
		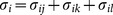
		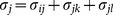
		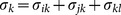
		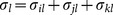
		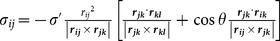
		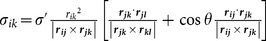
**Dihedral**	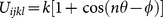	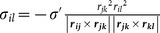
		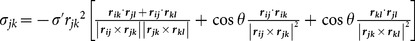
		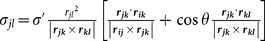
		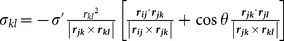
		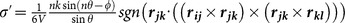
**Coulomb**	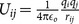	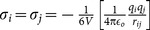
**vdW**	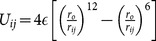	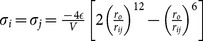
**GB (Implicit solvent)**	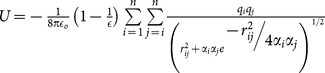	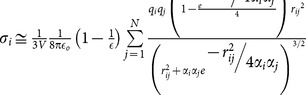

Although a formalism for including long-ranged electrostatic interactions in a periodic simulation has previously been described [27], it was reported to require about 30 minutes to process one trajectory frame of the small protein ubiquitin. While significant performances gains might be possible via carefully tuned code along with the use of GPUs, we find that the strongest forces, and hence the largest contributions to the stress, are short-ranged. It is therefore reasonable to omit long-ranged electrostatic contributions to the stress, especially given that many applications of CAMS will likely seek qualitative insight more than quantitative rigor. It is also worth noting that the use of bond-length constraints, by methods such as SHAKE [53], can lead to errors in the virial, unless special measures are taken. It is therefore recommended that, for applications where more quantitative results are sought, CAMS be applied to simulation data where bond-lengths are not constrained.

The tensor form (3×3 matrix) of stress poses problems for visualization and comparative analysis, as the tensor components vary with orientation, and it is cumbersome to visualize a multicomponent tensor at each atom of a large system, such as a protein. We therefore simplify the representation by averaging the principal stresses at each atom and applying a sign change to yield the local hydrostatic pressure. (Note that by convention, hydrostatic pressure has the opposite sign of the local stress, such that regions with negative hydrostatic pressure are under tension and regions with positive hydrostatic pressure are under compression.) No diagonalization is required to compute the average principal stress, as this quantity is simply one-third of the trace of the stress tensor. This eliminates the need to compute the off-diagonal stress tensor components and thus speeds the calculations while preserving the ability to distinguish between compression and tension. Furthermore, using the identity, *trace(A+B)  =  trace(A) + trace(B)*, one may obtain the total stress at an atom as the sum of contributions from the potential terms in an additive force-field, such as bond, angle, dihedral, van der Waals, Coulomb, and generalized Born. The current implementation of the software only supports computing the average of the principal stresses on each atom, and its decomposition, but it may be useful to write out the full stress tensor, with its off-diagonal elements, in a later version of the software.

The CAMS package reads in three files in the GROMACS [41], [42] format: an index file (*.ndx), a topology file (*.tpr), and a binary trajectory file (*.trr). If the system includes explicit solvent with periodic boundaries, then the trajectory coordinates need to be imaged/wrapped, with the solute centered in the simulation box, prior to running CAMS. An installation of GROMACS is also necessary to properly build all of the input files. The standard output comprises a data file containing the total stress per atom for each snapshot in the trajectory file for the set of atoms specified in the index file, along with four structure files in pdb format containing the input coordinates used to build the.tpr file, where the beta columns contain either the total stress per atom, the total stress per residue, the mean square fluctuation of the stress per atom, or the mean square fluctuation of the stress per residue. Additionally, the user may specify the “-split” flag to generate analogous output files for the individual stress contributions: bonds, angles, dihedral, Coulombic, solvent (GB), van der Waals, all nonbonded, all bonded, and kinetic (if the trajectory contains velocities). For simulations involving the AMBER software package [48], [54], we use the script amb2gmx.pl [55] along with the GROMACS tool 'grompp' to convert AMBER prmtop topology files into the GROMACS format. For netcdf (*.nc) trajectory files, we use VMD [56] to generate the GROMACS binary format (*.trr). Currently, this conversion process results in the removal of velocities from the netcdf trajectory if the trajectory contained the velocities. Future versions of the code are planned to contain native support for AMBER file formats.

### Software validation

We verified the CAMS software package in several ways. First, we checked that the forces computed via the CAMS software matched identically to the forces computed directly by GROMACS. This verifies that the CAMS software package correctly parses the coordinates, parameters, and topology of the structures, and that that the analytical forms of the gradients are correct. Second, we validated the computed stress values via a variety of simple test structures, up to 5 atoms in size, which are small enough that computing the associated stress values is tractable by hand. The test structures, topology, and stress output files have been added to the CAMS software package so that new users can check whether or not the software compiled correctly and is producing expected values.

### Applicability of CAMS stress software

The CAMS package can be used to compute stresses for a wide range of simulated molecular systems, with or without explicit solvent. The software supports any additive potential which uses the functional forms listed in Table 1. The 10 Å nonbonded cutoff and the solvent dielectric constant of 80 in the GB implementation are currently hard-coded, but these values can be changed through recompilation. Currently, the molecular topology and parameters must be available in the format of a GROMACS *.tpr file, and the coordinate trajectories as a binary *.trr file type, but the package includes tools for converting AMBER's prmtop/inpcrd files into these required GROMACS formats. It is worth noting that CAMS can be applied to simulations in which artificial external forces are applied to the molecular system, such as via steered MD or other external loading conditions.

### Molecular dynamics data

#### Bovine pancreatic trypsin inhibitor (BPTI)

Time-averaged stresses and stress fluctuations were computed for the longest, continuous MD simulation of a protein published to date, a 1 ms simulation of the small trypsin inhibitor BPTI [57]. The trajectory contains over 4 million snapshots separated into several conformational clusters [57]. Although the CAMS software can be used to compute stresses that account fully for the presence of explicit solvent, here we removed the explicit waters from the trajectory and used GB theory, instead, to estimate the solvent contributions to the atomic virial stress, in order to speed up the stress calculations. An identical protocol of replacing the explicit waters with implicit water for post processing analyses is commonly used when estimating free energy differences via MM-GBSA and should suffice when qualitatively investigating stress differences in structural features of the protein. We emphasize that this approach is only recommended for estimating differences in stress values when it is not critical to estimate those differences quantitatively. Stresses and stress fluctuations were compared between clusters 1 and 2, which have the largest differences in entropy and enthalpy [58] and a significant difference in the correlation of their respective configurational entropies [59]. These quantities are averaged by residue and displayed by color on a conformer representative of cluster 1.

#### Graphene nanostructures

We also studied the propagation of high-energy stress waves in graphene nanoribbons and nanotubes. The atomic geometries of these systems were constructed using the Carbon Nanostructure Builder plugin to VMD 1.9.1 [56]. All of the carbon atoms of C_60_, the nanoribbons, and the nanotubes were then set to be the atomtype “ca” to represent aromatic carbon with sp^2^ bond order for use with the Generalized Amber Force Field (GAFF) 1.4 [60], and all partial charges were set to zero. The AMBER12 software package [48] with GPU support [61], [62] was used to simulate these systems. First, the systems were energy-minimized with 1000 steps of steepest descent followed by up to 4000 steps of conjugate gradient such that the root-mean-square of the gradient is less than 0.0001 kcal/mole-Å. Next, the system was heated to 1 K over the course of 1000, 0.5 fs timesteps with all atoms restrained to their initial positions by a weak (1.0 kcal/mol/A^2^) harmonic restraint. The heating step generated a necessary restart file with an initial very small velocity assigned to each atom. The restart file was then manually edited to substitute in larger initial velocities (∼20.5 km/s or ∼204.5 A/ps) for selected atoms or components as detailed in the Results section, to initiate either a collision or a wave pulse. Finally, the system was allowed to evolve during an NVE simulation for 1.5 ps with 0.05 fs time steps. These short time-steps are required because of the high initial velocities. All carbon atoms were gently restrained by a 0.1 kcal/mol/A^2^ harmonic restraint during the production NVE simulations, in order to prevent overall translation of the graphene system. The small contributions from the very weak restraints were not included in the stress calculations as they would only play a minor role when visualizing the shock pulse as it traveled through the ultra cold graphene atoms.

## Results and Discussion

### Equilibrium stresses in BPTI

Like an engineered structure, a protein may be expected to have nonuniform distributions of internal stress, with potential implications for kinetic and thermodynamic stability as well as function. In addition, the temporal fluctuations of stress that result from thermal motion may provide insight regarding local elasticity. Here we report the computations of mean stress and stress fluctuations in BPTI.

**Figure 1:**
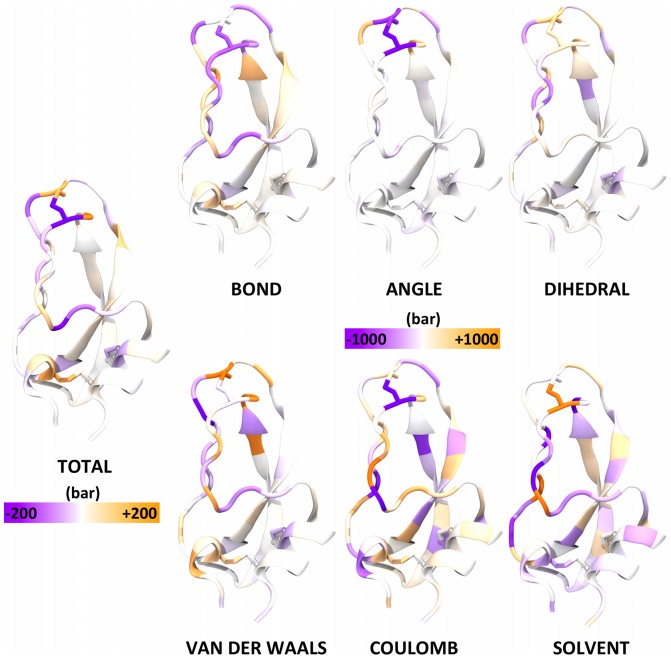
Residue-averaged differences in stress between clusters 1 and 2 (cluster 1 minus cluster 2). The left color spectrum applies to the total stress, and the right color spectrum applies to all of the stress components.

We focus in particular on differences in the atomic virial stresses averaged over all atoms within an amino acid residue between the two most thermodynamically distinct conformational clusters (1 and 2) identified in a 1 ms BPTI molecular dynamics simulation [57]–[59]. We compute the residue-averaged stress for residue 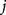 per snapshot 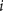 as, 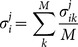
where 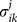 is the instantaneous stress of atom 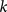 within residue 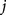, and 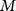 is the number of atoms per residue type. While such averaging results in stresses that in principle cannot be summed in a way to determine the virial of the whole system without properly shifting into the Lagrangian frame of reference of the residue, it can provide a clearer picture of the stress differences within distinct structural features, e.g. disulfide bridges, than the atomistic values. This residue-averaging approach provides an intuitive method of highlighting potentially mechanistically interesting regions within the structure. The differences in the total stress (Fig. 1, left) highlight a greater degree of tensile stress (purple), for cluster 1 relative to cluster 2, in the loop disulfide at the top of the protein (Cys14-Cys38), as well as in a segment of strand near the front, while other localized regions are under greater compressive stress (orange). Note that the loop disulfide has different preferred conformers in the two clusters. It is interesting to speculate that large stress differences may highlight residues that play key structural roles in stabilizing the two conformational states. In addition, differences in disulfide stress have been related to differences in chemical reactivity [63].

Individual stress components (Fig. 1, right) show larger differences (note change in color scale) than the total stress, indicating cancellation across terms. Not surprisingly, for example, differences in Coulombic stress are largely balanced by opposite changes in solvent-induced stress, computed with the GB model. Most of the tensile stress in the loop disulfide is observed to be associated with angle-bend terms, while compression at the lower left of the protein derives from both bond-stretch and van der Waals stresses.

**Figure 2:**
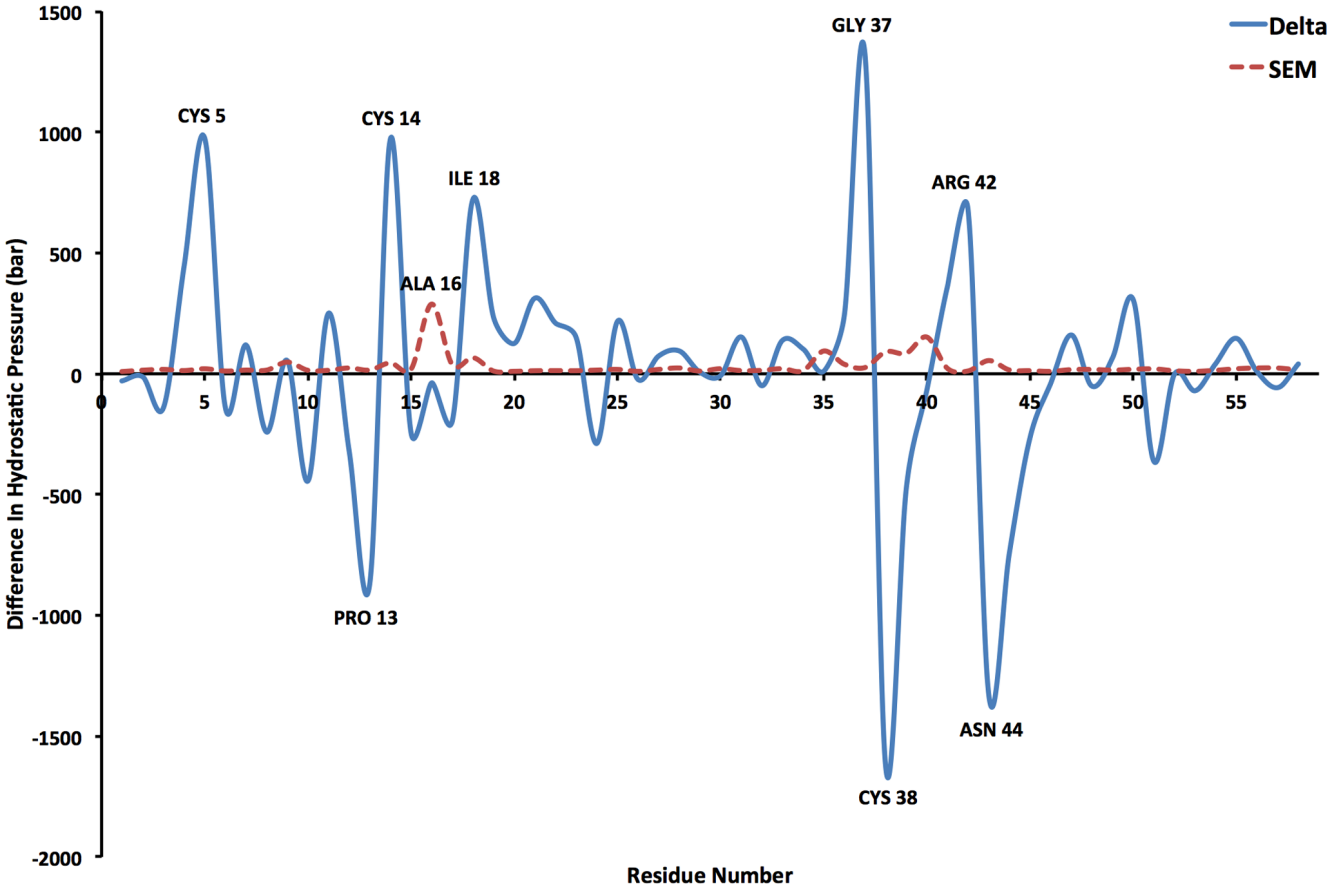
The delta in residue-averaged hydrostatic pressure between clusters 1 and 2 (solid, blue line) and the associated standard error of the mean (dashed, red line) for all 58 residues of BPTI. Residues with large (greater in magnitude than 500 bar) are labeled.

Estimates of the standard error of the mean (SEM) for the residue average stress values were computed using 'pymbar', a statistical software package capable of determining the statistical inefficiency within a time series of data [64]. We found the SEM values of cluster 1 to vary from 0.005 to 0.02 kbar, and those of cluster 2 to vary from 0.01 to 0.3 kbar. For a given residue, we combined the SEM values in quadrature when computing the differences in residue-averaged stresses. The combined SEM values associated with the delta between clusters ranged from 0.009 to 0.3 kbar. The delta in residue-averaged hydrostatic pressure between the two clusters per residue and the associated combined SEM values are shown in Fig. 2.

**Figure 3:**
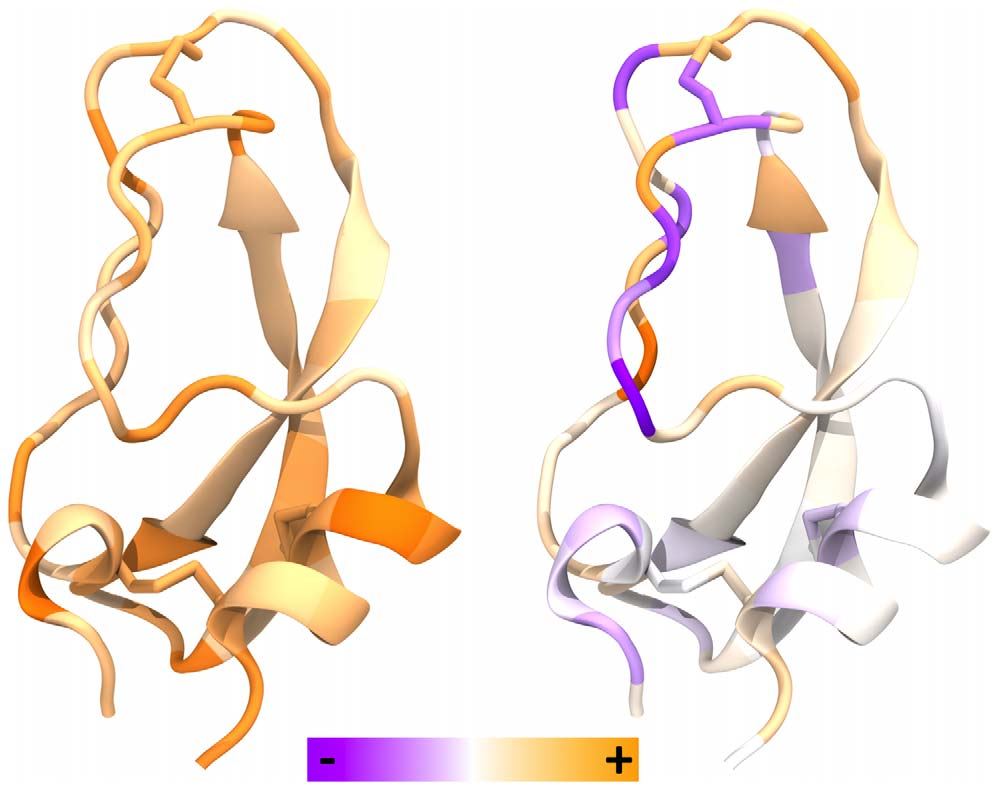
Mean square fluctuations of the residue-averaged stresses computed from the 1 ms BPTI trajectory. (Left) Cluster 2; values range from 1.50 to 5.08 Mbar. (Right) Difference between cluster 1 and 2 (cluster 1 minus cluster 2); values range from −90.3 to 63.6 kbar. Purple (negative) and orange (positive) indicate regions where cluster 1 has less or more stress fluctuations than cluster 2, respectively.

We compute the mean square fluctuation (MSF) of the total residue-averaged stress per residue 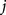 as,



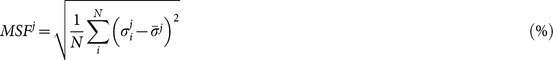
where 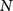 is the number of snapshots, 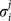 is total stress for residue 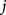 at snapshot 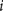, and 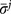 is the total residue-averaged stress over the whole trajectory for residue 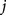. Fig. 3 (left) shows the MSF values for all residues when BPTI is in conformational cluster 2; the corresponding result for cluster 1 looks the same, as the differences in the MSF values are small relative to the absolute values, and therefore is not shown. The distribution of stress fluctuations is quite heterogeneous, with larger fluctuations in the lower part of the protein, whose conformational fluctuations are relatively modest and which contains alpha helices, which may be expected to be relatively stiff. On the other hand, the more flexible loop region at the top of the protein shows smaller stress fluctuations. Differences in stress fluctuations between the relatively rigid cluster 1 and more flexible cluster 2 are displayed in the right-hand side of Fig. 3. Although the largest differences are roughly two orders of magnitude less than the total values (∼10^3^ kbar^2^ vs. ∼10 kbar^2^), they clearly highlight the loop region of the protein, which is the part whose structure and dynamics differs most between the two clusters. Although cluster 1 is more rigid than cluster 2 [58], regions of both increased and decreased stress fluctuations are observed.

### Stress waves in graphene nanostructures

Pure carbon materials, e.g. graphene, can form a wealth of different structures at various length scales and geometries, yielding a large range in mechanical and electronic material properties [65]. These materials have a variety of uses, for example, ion beams of charged fullerenes at energies greater than 10 keV are used in time-of-flight secondary ion mass spectrometry [66], while graphene has many potential applications including transistors [67], filters for desalination [68], and supercapacitors [69], [70]. Here, we use CAMS to visualize waves generated by large mechanical perturbations, such as collisions, in several different graphene constructs.

**Figure 4:**
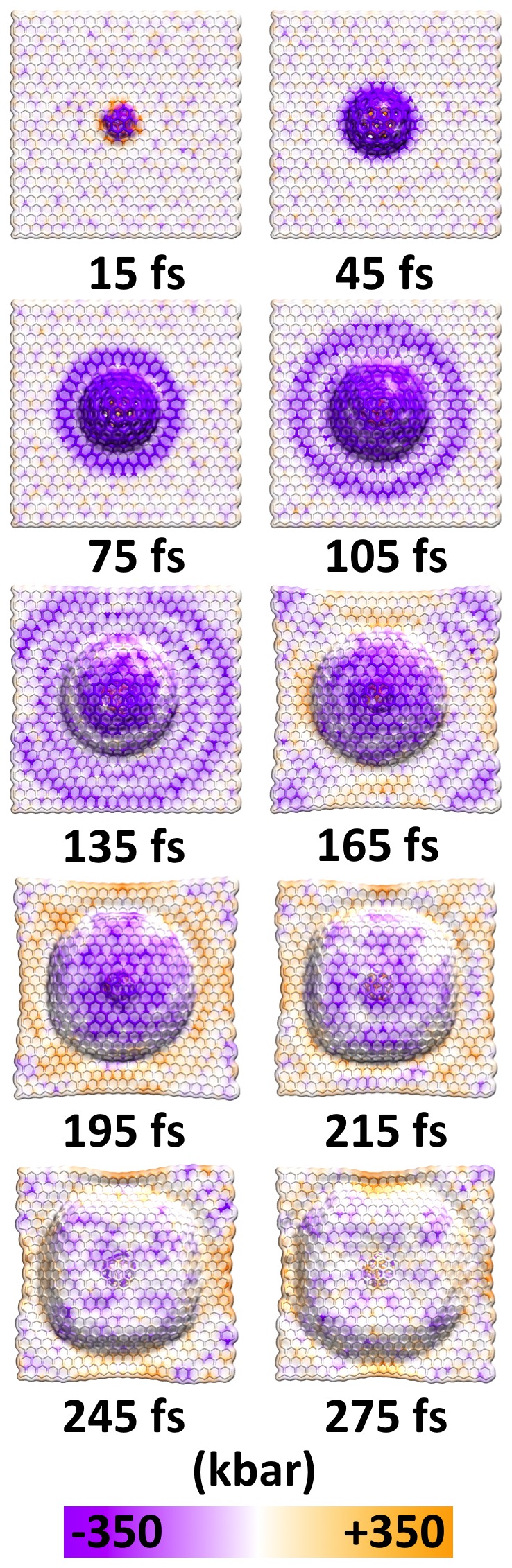
Time series of wave propagation through a monolayer of graphene after the impact of a hypervelocity fullerene. The passage of time is measured relative to the point of impact. After the initial collision, longitudinal stress waves (purple tensile band) propagate radially outward at a greater velocity than the transverse deformation wave. Within 165 fs since the moment of impact, regions of the longitudinal wavefront reflected (orange compressive regions) at the boundaries and headed towards the wavefront of the transverse deformation wave. Nonuniform interaction between the two waves has distorted the spherical transverse deformation wave.

First, we investigated stress waves in a monolayer of graphene initiated by the impact of a hypervelocity C_60_ fullerene (∼20.5 km/s; 1.8 keV) [66], [71]. Fig. 4 shows the time-evolution of the waves from the moment of impact. Initially, radially symmetric longitudinal tensile waves (colored purple in Fig. 4) rapidly spread out from the point of impact, moving at ∼12 km/s, which is just over half the experimental speed of sound in graphene (21 km/s) [72], [73]. A transverse wave, traveling at ∼7 km/s, lags the longitudinal waves as the collision visibly deforms the graphene sheet out of its plane. The reflection of the longitudinal wave from the edge of the sheet results in compression (orange in Fig. 4) at the edges of the graphene monolayer and interacts with the leading edge of the transverse wave. The collision of the two wavefronts impedes regions of the transverse wave and thus alters the shape of the transverse wavefront. Visualization of the resulting tensile and compressive stresses as the waves propagate throughout the material clearly highlights the shapes and interaction regions of the waves. These reported pressures, shown in Fig. 4, are within the tolerance of the material, as graphene has been measured to have an intrinsic (ultimate tensile) strength of 1.3 Mbar [74].

**Figure 5:**
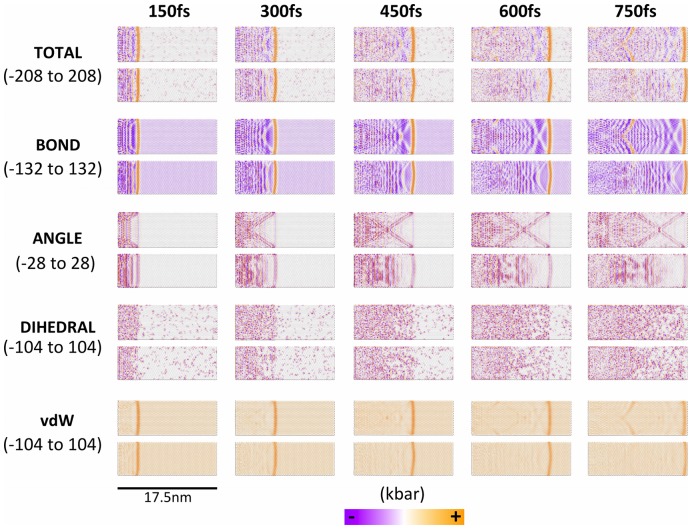
Stress decomposition of a wave pulse traveling left to right through graphene nanoribbons either in the armchair (top row of each pair) or zigzag (bottom row of each pair) configurations.

Next, we investigated wave propagation through graphene nanoribbons by applying a 23 km/s velocity pulse uniformly to an edge of the nanoribbon, where the carbons are either in the “zigzag” or “armchair” configuration [65]. This resulted in propagation of a sharply defined pressure wave along the nanoribbon, with a trailing pattern of excitations that are clearly visualized by the color-coded atomistic stresses, as illustrated for a series of time-points in Fig. 5. The main wave-front is slightly curved, suggesting a somewhat slower velocity at the edges of the ribbon. Interestingly, although the configuration of the ribbon (zigzag vs. armchair) does not greatly affect the shape and velocity of the total stress wavefront (Fig. 5, top row), decomposition of the stresses into bonded and nonbonded contributions showed striking differences and emergent patterns in some of the contributions (Fig. 5, lower 4 rows). In particular, the stresses resulting from the bond and angle terms show distinct patterns in the region of the nanoribbons behind the wavefront, including an “X” configuration of angle stresses in the armchair configuration, which is absent in the zigzag configuration. There are also clear distinctions between the two nanoribbon configurations in the bond and van der Waals stresses.

**Figure 6:**
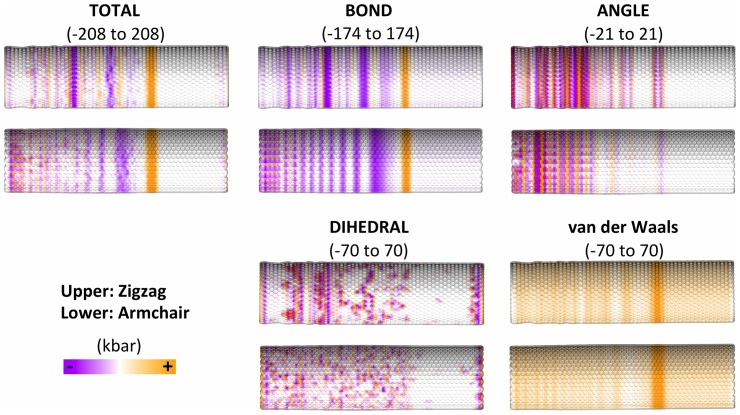
Stress decomposition of a wave pulse traveling left to right through graphene nanotubes either in the armchair (upper) or zigzag (lower) configurations. Data are shown for the 450 fs time-point.

In order to determine which of the patterns observed in the nanoribbons (Fig. 5) resulted from edge effects, we performed the same analysis on graphene nanotubes, where edge effects are absent. Fig. 6 shows that, while the leading wavefront from the initial pulse is no longer slowed down by the edges, there are now far more uniform trailing stress waves of opposite sign and in different locations depending on the carbon configurations. The bond stresses are the primary origin of these bands, and the differences in the angle stresses between the two carbon configurations are less striking in the nanotubes than the nanoribbons. Therefore, edge effects seem to play a major role in the propagation and dispersion of stress waves in graphene sheets.

## Conclusions

We have described CAMS, a new software package, which reads in a molecular dynamics trajectory and associated parameters files, and writes out trajectory frames annotated with atomistic virial stresses, including contributions from non-central force terms. The output includes not only the total stress, but also the stress contribution from each term in the potential function. Mean stresses and stress fluctuations computed for an equilibrium simulation of BPTI show heterogeneous patterning that correlates with structural elements and with conformational changes and may help to highlight residues playing key structural or functional roles. The propagation of stress waves in graphene nanostructures is clearly visualized as well, enabling identification of edge effects, wave dispersion, and distinct contributions of the various terms in the potential function. Fruitful applications are expected in studies of protein allostery, DNA coiling and packing, molecular machines, and mechanosensors. The CAMS package can also be applied to simulations in which external forces are applied to a protein, such as via steered molecular dynamics or the addition of artificial springs, thus enabling mechanical studies of biomolecules.

The CAMS source code has been deposited at the stable GitHub repository http://github.com/afenley/CAMS, where it is available for general use and further development under a GNU General Public License. Topology information for the graphene and BPTI systems along with the residue-averaged stresses for BPTI are included with the source code. The repository also contains test cases, and a tutorial for using CAMS with the AMBER biomolecular simulation package is planned. Potential future developments include the provision of command-line access to additional parameters, such as the nonbonded cutoff radius; calculation and output of off-diagonal stress tensor terms; and the ability to handle additional force field functional forms, such as ones that allow bond-breaking and bond-making.
